# Effect of the COVID-19 Pandemic on Treatment Delays in Patients with ST-Segment Elevation Myocardial Infarction

**DOI:** 10.3390/jcm9072183

**Published:** 2020-07-10

**Authors:** Sebastian J. Reinstadler, Martin Reindl, Ivan Lechner, Magdalena Holzknecht, Christina Tiller, Franz Xaver Roithinger, Matthias Frick, Uta C. Hoppe, Peter Jirak, Rudolf Berger, Georg Delle-Karth, Elisabeth Laßnig, Gert Klug, Axel Bauer, Ronald Binder, Bernhard Metzler

**Affiliations:** 1 University Clinic of Internal Medicine III, Cardiology and Angiology, Medical University of Innsbruck, Anichstrasse 35, A-6020 Innsbruck, Austria; sebastian.reinstadler@tirol-kliniken.at (S.J.R.); Martin.Reindl@tirol-kliniken.at (M.R.); Ivan.Lechner@tirol-kliniken.at (I.L.); Magdalena.Holzknecht@tirol-kliniken.at (M.H.); Christina.Tiller@tirol-kliniken.at (C.T.); Gert.Klug@tirol-kliniken.at (G.K.); Axel.Bauer@tirol-kliniken.at (A.B.); 2 Department of Cardiology, Wiener Neustadt Hospital, A-2700 Wiener Neustadt, Austria; FranzXaver.Roithinger@wienerneustadt.lknoe.at; 3 Department of Cardiology, Academic Teaching Hospital Feldkirch, A-6800 Feldkirch, Austria; matthias.frick@vlkh.net; 4 Clinic of Internal Medicine II, Department of Cardiology, Paracelsus Medical University of Salzburg, A-5020 Salzburg, Austria; u.hoppe@salk.at (U.C.H.); p.jirak@salk.at (P.J.); 5 Department of Cardiology and Internal Medicine, Hospital of St. John of God, A-7000 Eisenstadt, Austria; Rudolf.Berger@bbeisen.at; 6 Department of Cardiology, Vienna North Hospital, A-1210 Vienna, Austria; georg.delle-karth@wienkav.at; 7 Department of Cardiology and Intensive Care, Klinikum Wels, A-4600 Wels, Austria; elisabeth.lassnig@klinikum-wegr.at (E.L.); Ronald.Binder@klinikum-wegr.at (R.B.)

**Keywords:** Coronavirus disease 2019, ST-segment elevation myocardial infarction, total ischemic time

## Abstract

Coronavirus disease 19 (COVID-19) and its associated restrictions could affect ischemic times in patients with ST-segment elevation myocardial infarction (STEMI). The objective of this study was to investigate the influence of the COVID-19 outbreak on ischemic times in consecutive all-comer STEMI patients. We included consecutive STEMI patients (*n* = 163, median age: 61 years, 27% women) who were referred to seven tertiary care hospitals across Austria for primary percutaneous coronary intervention between 24 February 2020 (calendar week 9) and 5 April 2020 (calendar week 14). The number of patients, total ischemic times and door-to-balloon times in temporal relation to COVID-19-related restrictions and infection rates were analyzed. While rates of STEMI admissions decreased (calendar week 9/10 (*n* = 69, 42%); calendar week 11/12 (*n* = 51, 31%); calendar week 13/14 (*n* = 43, 26%)), total ischemic times increased from 164 (interquartile range (IQR): 107–281) min (calendar week 9/10) to 237 (IQR: 141–560) min (calendar week 11/12) and to 275 (IQR: 170–590) min (calendar week 13/14) (*p* = 0.006). Door-to-balloon times were constant (*p* = 0.60). There was a significant difference in post-interventional Thrombolysis in myocardial infarction (TIMI) flow grade 3 in patients treated during calendar week 9/10 (97%), 11/12 (84%) and 13/14 (81%; *p* = 0.02). Rates of in-hospital death and re-infarction were similar between groups (*p* = 0.48). Results were comparable when dichotomizing data on 10 March and 16 March 2020, when official restrictions were executed. In this cohort of all-comer STEMI patients, we observed a 1.7-fold increase in ischemic time during the outbreak of COVID-19 in Austria. Patient-related factors likely explain most of this increase. Counteractive steps are needed to prevent further cardiac collateral damage during the ongoing COVID-19 pandemic.

## 1. Introduction

Coronavirus disease 2019 (COVID-19), caused by severe acute respiratory syndrome coronavirus 2 (SARS-CoV-2), was declared a pandemic on 11 March 2020 and caused a public health crisis of global proportions. Austria, with its ~8.8 million inhabitants, was one of the European countries affected early by COVID-19. The government executed the first large-scale public health measures on 10 March 2020, followed by a nationwide lockdown and quarantining of complete regions on 16 March 2020.

Although awareness of the natural history and associated morbidity and mortality of COVID-19 is growing rapidly [1,2,3,4,5,6], we have only scant data on the effects of COVID-19 on established medical care systems. This gap in knowledge also applies to the management of patients suffering from ST-segment elevation myocardial infarction (STEMI) [7], a very frequent cardiac emergency that significantly contributes to the global burden of cardiovascular disease [8,9]. The well-known time-dependent extension of myocardial necrosis is a major contributor to STEMI-related morbidity and mortality [8,10,11,12]. A first preliminary case series from Hong Kong with seven consecutive STEMI patients suggests longer ischemic times since the COVID-19 outbreak [13]. However, to our knowledge, the impact of COVID-19 on clinical characteristics and ischemic times of patients suffering from STEMI has not been systematically studied. To address this important lack of data, we aimed to examine the impact of COVID-19 on clinical characteristics and ischemic times in consecutive all-comer STEMI patients referred to percutaneous coronary intervention (PCI) centres throughout Austria.

## 2. Methods

### 2.1. Study Design and Population

This multicenter retrospective observational study was a collaborative effort between seven PCI centers in Austria during the COVID-19 outbreak. Together, the hospitals have a catchment area of about 2.2 million inhabitants, which is a quarter of the entire Austrian population. All consecutive patients admitted for STEMI among the centers in the network between 24 February 2020 and 5 April 2020 (calendar weeks 9 to 14) were included. STEMI was diagnosed in accordance with the current guidelines as the presence of clinical symptoms of myocardial infarction with specific STEMI-defining electrocardiographic criteria for >20 min [14]. The following electrocardiographic criteria were applied: ST-segment elevation in at least two contiguous leads, with ST-elevation ≥2 mm in men (≥2.5 mm in men <40 years) or ≥1.5 mm in women in leads V2-V3 and/or ≥1.5 mm in other leads [14]. All patients included in the present study were treated by primary PCI and received optimal medical therapy post-PCI as recommended by contemporary guidelines [14]. Patients treated with fibrinolysis were excluded.

The ethics committee approved the study and due to the nature of retrospective chart review, waived the requirement for informed consent from individual patients.

### 2.2. Data Collection and Study Objectives

All data were collected by the authors, who subsequently reviewed, analyzed and interpreted the results. All authors approved the final manuscript and vouch for the accuracy and completeness of the data and for the adherence to the study protocol. In case of missing data, requests for clarification were sent to the respective center.

The primary study objective was to assess possible differences in total ischemic time in patients suffering from STEMI during the COVID-19 outbreak in Austria. For this purpose, patients were grouped according to calendar weeks: group 1 (calendar weeks 9 and 10), group 2 (calendar weeks 11 and 12) and group 3 (calendar weeks 13 and 14). Moreover, additional timelines for analysis were the first official restrictive public health measures introduced on 10 March 2020, and the nationwide lockdown on 16 March 2020.

We further extracted demographic and clinical characteristics, angiographic findings and the documented peak troponin level during index hospitalization. Major cardiovascular risk factors (diabetes mellitus, hypercholesterolemia, hypertension, smoking status) and angiographic parameters (culprit lesion location, pre- and post-interventional Thrombolysis in myocardial infarction (TIMI) flow) were evaluated.

All-cause death during index hospitalization was assessed. Criteria for myocardial re-infarction were: (a) new ST-elevation ≥1 mm or new pathognomic Q waves in at least two contiguous leads with additional diagnostic evaluation (troponin levels), or (b) reoccurrence of clinical signs or symptoms following the initial event with a >20% increase in troponin level [15].

### 2.3. Statistical Analysis

Continuous variables are shown as median and interquartile ranges (IQRs). Categorical variables are expressed as numbers with corresponding percentages. To evaluate differences in continuous variables between groups, Mann–Whitney U test or Kruskal–Wallis test were applied as appropriate. Differences in categorical variables between groups were assessed by Chi-square test. A *p*-value of <0.05 was considered statistically significant for all tests. No sample size calculation was performed a priori, and sample size was equal to the number of STEMI patients admitted and treated during the study period. No imputation was made for missing data. SPSS Statistics 25.0 (IBM, Armonk, NY, USA) as well as MedCalc Version 15.8 (Ostend, Belgium) were used for statistical analyses. All statistics should be interpreted as descriptive.

## 3. Results

### 3.1. Demographic and Clinical Characteristics

**Figure 1:**
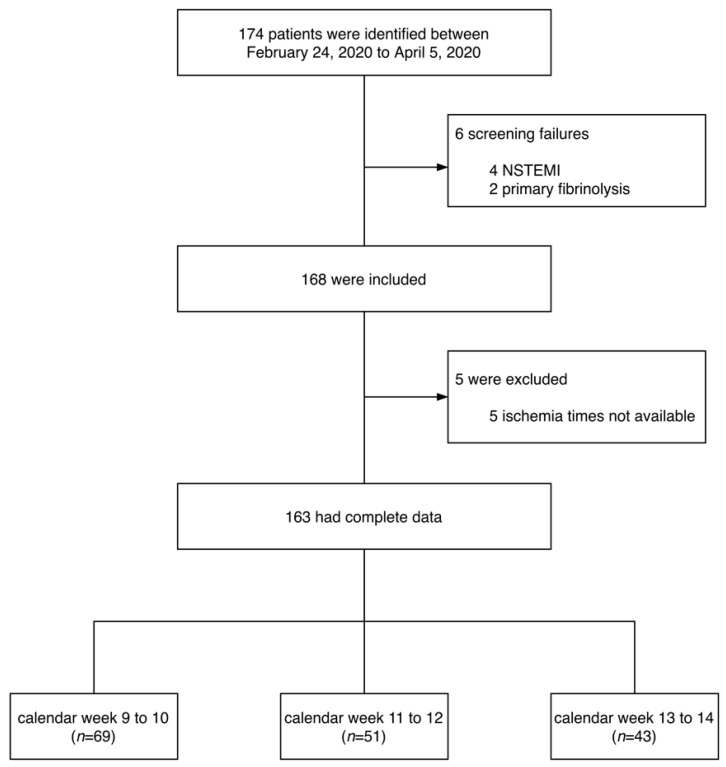
Flow chart of the study. NSTEMI = non-ST-elevation myocardial infarction.

**Figure 2:**
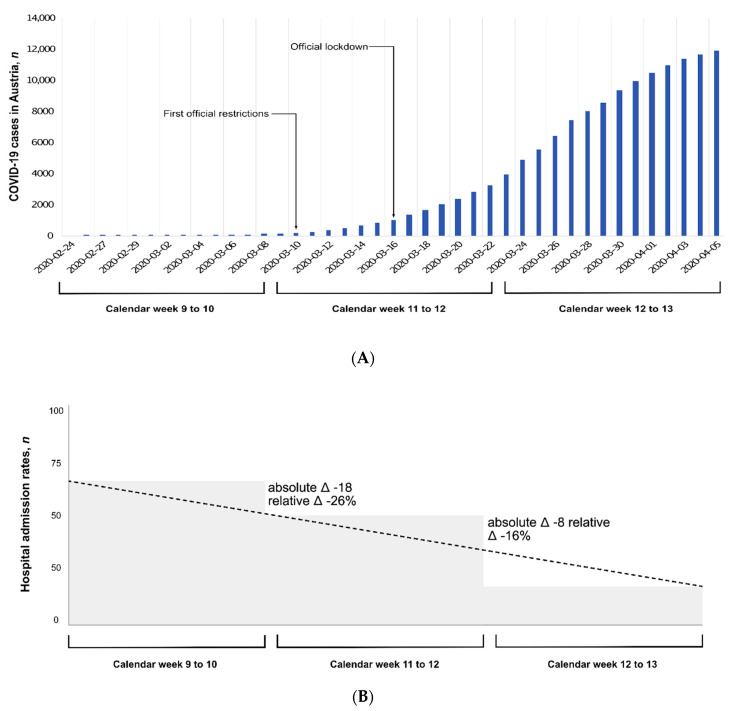

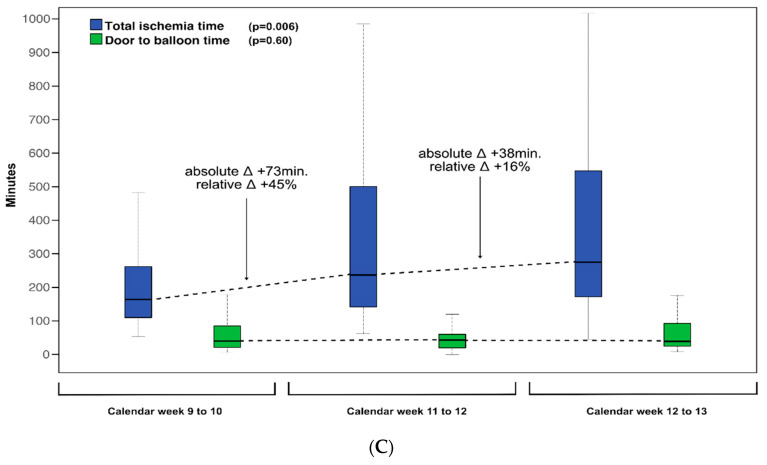
COVID-19 hospital admissions for STEMI and treatment delays. (**A**) Official statistics of all documented laboratory-confirmed cases of COVID-19 in Austria during the study period; (**B**) number of STEMI admissions compared between calendar week 9 and 10 (group 1), calendar week 11 and 12 (group 2), and calendar week 13 and 14 (group 3); (**C**) total ischemic time and door-to-balloon time of STEMI patients treated in calendar week 9 and 10 (group 1), calendar week 11 and 12 (group 2), and calendar week 13 and 14 (group 3). Abbreviations: STEMI = ST-elevation myocardial infarction; COVID-19 = coronavirus disease 2019.

One hundred and sixty-three patients admitted for primary PCI for STEMI between 24 February 2020 and 5 April 2020 were included (Figure 1). The demographic and clinical characteristics of the overall cohort are presented in Table 1. The characteristics according to calendar weeks (group 1: calendar week 9 to 10; group 2: calendar week 11 to 12; group 3: calendar week 13 to 14) are summarized in Table 2. The median age of the overall cohort was 61 (IQR: 55–74) years and 27% were women. Median total ischemic time was 201 (IQR: 130–405) min and increased from 164 (IQR: 107–281) to 237 (IQR: 141–560) and to 275 (IQR: 170–590) min from group 1 to group 3, respectively (*p* = 0.006). Door-to-balloon times did not differ significantly between groups (group 1: 40 (IQR: 21–85) min; group 2: 43 IQR: 20–60) min; group 3: 39 (IQR: 24–94) min; *p* = 0.60). The association between admission time point and treatment delays is illustrated in Figure 2. Other demographic characteristics did not significantly change during the study time.

Patient characteristics of those treated before (*n* = 72, 44%) and since (*n* = 91, 56%) 10 March 2020 (execution of first nationwide public health restrictions by the Austrian government) are summarized in Supplementary Table S1. Patient characteristics of those treated before (*n* = 100, 61%) and since (*n* = 63, 39%) 16 March 2020 (execution of a nationwide lockdown by the Austrian government) are summarized in Supplementary Table S2. The differences in the primary endpoint were the same as in the primary analysis.

### 3.2. Angiographic and Troponin Findings

The angiographic and biomarker findings according to calendar weeks are summarized in Table 2. There was a significantly lower post-PCI TIMI flow in patients treated during calendar week 11 to 12 (84%) and calendar week 13 to 14 (81%) compared with calendar week 9 to 10 (97%, *p* = 0.02). Median peak troponin levels increased by as much as a factor of 271 compared with the upper limit of normal; however, there was no significant difference between time groups (*p* = 0.97)

### 3.3. Adverse Events

The date of final clinical follow-up was 7 April 2020 and it was restricted to index hospitalization. Ten patients had intrahospital clinical events, including two (1%) myocardial re-infarctions and eight (5%) deaths. Five events (four deaths, one re-infarction) occurred in group 1 (7.2%), four events (three deaths, one re-infarction) occurred in group 2 (7.8%), and one event (one death) occurred in group 3 (2.3%) (*p* = 0.48). The event rate did not differ significantly when dichotomizing at 10 March or 16 March, 2020 (*p* = 0.70, *p* = 0.56, respectively).

## 4. Discussion

During the initial phase of the COVID-19 outbreak in Austria, the total ischemic time of STEMI patients admitted for primary PCI gradually increased from calendar weeks 9/10 to calendar weeks 13/14 across the country. Compared with the first two calendar weeks, patients admitted in the last two calendar weeks had 1.7-fold longer total ischemic times. Door-to-balloon times were similar during the study and therefore do not explain the increase in total ischemic times. The longer treatment delays were associated with lower PCI efficacy as defined by post-PCI TIMI flow. There were also fewer STEMI admissions at calendar weeks 11/12 (31%) and 13/14 (26%) compared to calendar weeks 9/10 (42%). Together, our observation from seven PCI centers across Austria with a catchment area of 2.2 million citizens (a quarter of the entire population of Austria) implies the need for proactive steps to mitigate cardiac collateral damage during the COVID-19 pandemic caused by longer total ischemic times in patients suffering from STEMI.

The first and only case series evaluating the impact of the COVID-19 outbreak on STEMI care described changes in time components of STEMI among seven consecutive patients who underwent primary PCI at Queen Mary Hospital in Hong Kong [13]. The authors observed longer median times in all components, with largest the differences in patient-related delays. Our multicenter study significantly expands these findings by showing up to 1.7 times longer total ischemic times in a large cohort of consecutive all-comer patients admitted and treated for acute STEMI after the COVID-19 outbreak in Austria. This alarming finding clearly indicates cardiac collateral damage as a result of COVID-19, as it is well-known that the best therapy for STEMI is a fast mechanical restoration of coronary blood flow with the placement of a stent [16]. The exact reasons for this observation are likely multifactorial and cannot be addressed by our retrospective study, but fear of infection is probably a major driver for delayed presentations. Patient-related delays likely explained the longer total ischemic time in our study, underscoring this hypothesis. In contrast, in our observation including seven PCI centers that are all embedded in regional STEMI care networks, there was no difference in door-to-balloon times. These findings suggest that patient-related but not system-related delays are of major relevance during the COVID-19 pandemic. Immediate efforts to reduce total ischemic time should therefore primarily focus on patient-related factors to reduce the medical and social effects of worsened STEMI management. Educational and promotional strategies are needed to raise the awareness of STEMI symptoms and the importance of urgent hospital admission among the general public, especially in times of a pandemic.

Patients who do not get successful reperfusion are at higher risk of complications and death. Along with increased total ischemic time, we observed lower rates of post-PCI TIMI flow grade 3, a widely used marker of reperfusion success with prognostic implications [17,18]. These observations underscore once again that efforts should be made to obtain early and thus optimal restoration of culprit vessel blood flow. In this context of prolonged ischemic times, the therapeutic option of pre-hospital fibrinolysis has to be discussed. In our analysis at the beginning of the COVID-19 outbreak in Austria, patient-related aspects were identified as driving factors for prolonged total ischemic times [19]. Current guidelines recommend the system-delay-based 120 min cut-off as the anticipated reperfusion time (from STEMI diagnosis to wire crossing) to decide on a primary PCI or fibrinolysis strategy [14]. However, in a clinical scenario of systematically prolonged ischemic times due to patient delays, facilitated PCI by pre-hospital fibrinolysis may be a potential therapeutic option [20,21,22,23]. In several countries, the COVID-19 pandemic has also affected health care structures and systems, leading to extended system delays. In the case of such system-related delays, the role of pre-hospital fibrinolysis has to be emphasized [24].

Despite the described association between acute respiratory infections and myocardial infarctions [25], we found a decline in the number of STEMI admissions during our study. While 42% presented during the first two calendar weeks, only 31% and 26% presented during calendar weeks 11/12 and 13/14, respectively. This decline in STEMI admissions and thus treatment of the disease with the beginning of the COVID-19 pandemic is another factor that supports the hypothesis that patients with cardiovascular complaints come late or may even not seek medical help at all [26,27]. The decrease in hospital admissions due to STEMI may, however, also be partly explained by a reduction in myocardial infarction triggers [27].

Our study has several strengths. First, it provides the first comprehensive estimate on the impact of the COVID-19 outbreak on treatment delays in consecutive all-comer STEMI patients treated with contemporary primary PCI. Considering the catchment area of 2.2 million inhabitants, a STEMI incidence rate of 95 per 100,000 per year in Austria [28] and taking into consideration sudden cardiac deaths (~20%) [29], we would theoretically expect 182 admitted STEMI patients for the period of this study. This fits well with the observed STEMI incidence in our analysis. Second, our study included patients that were treated within modern STEMI networks from seven centers across the whole country, which further underscores the generalizability of our findings. Despite these unique strengths, our study also has important limitations. First, this was a retrospective study and data generation was therefore clinically driven and not systematic. Hence, comprehensive imaging data and non-routine biomarkers (e.g., natriuretic peptides) cannot be provided. The results of this time-series analysis should therefore be interpreted with caution, as confounders could not be excluded. However, there is an urgent need for knowledge in this worldwide crisis without precedent. Few cases had incomplete documentation of clinical characteristics and ischemic times (the latter were excluded from the analysis). The observed data on clinical outcomes should be considered exploratory only, as the present study was not powered to assess differences in this endpoint. Finally, follow-up was restricted to the index hospital stay.

## 5. Conclusions

In conclusion, the observations described here show clinically relevant longer total ischemic times among patients suffering STEMI after the outbreak of COVID-19. The increase of total ischemic times was not explained by the door-to-balloon time, which was similar at all time points. Lessons learned from this observation may provide useful information for health care systems and authorities all over the world. This information may contribute to minimize cardiac collateral damage during the rapidly spreading COVID-19 pandemic.

## Figures and Tables

**Table 1:** Patient characteristics of the overall study population.

Characteristic	Total Population (*n* = 163)
Age, years (IQR)	61 (55–74)
Female, *n* (%)	44 (27)
Body weight, kg (IQR)	81 (70–90)
Height, cm (IQR)	174 (168–180)
Body mass index, kg/m^2^ (IQR)	26.7 (24.2–29.8)
Diabetes mellitus, *n* (%)	32 (20)
Current smoker, *n* (%)	71 (44)
Hypercholesterolemia, *n* (%)	132 (81)
Hypertension, *n* (%)	103 (63)
Previous myocardial infarction, *n* (%)	21 (13)
Previous diagnosis of CCS, *n* (%)	34 (21)
Previous CABG, *n* (%)	5 (3)
Heart rate, bpm (IQR)	73 (62–91)
Sinus rhythm, *n* (%)	146 (90)
Systolic blood pressure, mmHg (IQR)	130 (110–150)
Diastolic blood pressure, mmHg (IQR)	75 (64–90)
Killip class >I, *n* (%)	50 (31)
Peak troponin, x-fold increase of ULN (IQR)	271 (108–578)
Total ischemic time, min (IQR)	201 (130–405)
Door-to-balloon time, min (IQR)	40 (21–81)
Culprit lesion, *n* (%)	
RCA	58 (36)
LAD	78 (48)
LCX	19 (12)
RI	2 (1)
LM	5 (3)
Bypass graft	1 (1)
Pre-interventional TIMI flow grade 0, *n* (%)	95 (58)
Post-interventional TIMI flow grade 3, *n* (%)	145 (89)

Abbreviations: CCS = chronic coronary syndrome; CABG = coronary artery bypass graft; ULN = upper limit of normal; RCA = right coronary artery; LAD = left anterior descending artery; LCX = Left circumflex artery; RI = ramus intermedius; LM = left main coronary artery; TIMI = thrombolysis in myocardial infarction; IQR = interquartile range.

**Table 2:** Differences in patient characteristics during the COVID-19 outbreak.

	Calendar week 9–10 (*n* = 69, 42%)	Calendar week 11–12 (*n* = 51, 31%)	Calendar week 13–14 (*n* = 43, 26%)	*p*-Value
Age, years (IQR)	61 (54–72)	63 (55–76)	60 (56–71)	0.60
Female, *n* (%)	21 (30)	12 (24)	11 (26)	0.68
Body weight, kg (IQR)	80 (70–90)	80 (70–90)	85 (73–99)	0.26
Height, cm (IQR)	174 (165–180)	176 (169–180)	172 (166–178)	0.26
Body mass index, kg/m^2^ (IQR)	26.3 (24.2–29.5)	26.1 (23.6–28.7)	28.1 (24.4–31.8)	0.11
Diabetes mellitus, *n* (%)	13 (19)	8 (16)	11 (26)	0.44
Current smoker, *n* (%)	27 (39)	24 (47)	20 (47)	0.63
Hypercholesterolemia, *n* (%)	55 (80)	40 (78)	37 (86)	0.46
Hypertension, *n* (%)	45 (65)	31 (61)	27 (63)	0.85
Previous myocardial infarction, *n* (%)	8 (12)	8 (16)	5 (12)	0.67
Previous diagnosis of CCS, *n* (%)	16 (23)	13 (25)	5 (12)	0.31
Previous CABG, n (%)	2 (3)	2 (4)	1 (2)	0.91
Heart rate, bpm (IQR)	74 (65–90)	70 (60–85)	78 (60–97)	0.52
Sinus rhythm, *n* (%)	59 (86)	47 (92)	40 (93)	0.48
Systolic blood pressure, mmHg (IQR)	135 (108–151)	127 (108–140)	130 (114–152)	0.44
Diastolic blood pressure, mmHg (IQR)	74 (62–90)	75 (65–89)	80 (64–94)	0.64
Killip class > I, *n* (%)	20 (29)	16 (31)	14 (33)	0.85
Peak troponin, x-fold increase of ULN (IQR)	268 (138–580)	295 (85–636)	272 (108–255)	0.97
Total ischemic time, min (IQR)	164 (107–281)	237 (141–560)	275 (170–590)	0.006
Door-to-balloon time, min (IQR)	40 (21–85)	43 (20–60)	39 (24–94)	0.60
Culprit lesion, *n* (%)				0.34
RCA	23 (33)	18 (35)	17 (40)	
LAD	35 (51)	23 (45)	20 (46)	
LCX	9 (13)	6 (12)	4 (9)	
RI	0 (0)	0 (0)	2 (5)	
LM	2 (3)	3 (6)	0 (0)	
Bypass graft	0 (0)	1 (2)	0 (0)	
Pre-interventional TIMI flow 0, *n* (%)	39 (57)	28 (55)	28 (65)	0.60
Post-interventional TIMI flow 3, *n* (%)	67 (97)	43 (84)	35 (81)	0.02

Abbreviations: CCS = chronic coronary syndrome; CABG = coronary artery bypass graft; ULN = upper limit of normal; RCA = right coronary artery; LAD = left anterior descending artery; LCX = Left circumflex artery; RI = ramus intermedius; LM = left main coronary artery; TIMI = thrombolysis in myocardial infarction; interquartile range (IQR).

## Supplementary Materials

The following are available online at https://www.mdpi.com/2077-0383/9/7/2183/s1, Table S1: Differences in clinical characteristics before and since 10 March 2020, Table S2: Differences in clinical characteristics before and since 16 March 2020.
