# Supplementary material for: Effect of the COVID-19 Pandemic on Treatment Delays in Patients with ST-Segment Elevation Myocardial Infarction
Source: J Clin Med. 2020 Jul 10;9(7):2183. doi: 10.3390/jcm9072183 (PMC7408681; doi:10.3390/jcm9072183)
Supplement: Supplementary file 1 [file jcm-09-02183-s001.pdf]

eTable 1: Differences in clinical characteristics before and since March 10, 2020

	Before March 10, 2020 (n=72, 44%)	From March 10, 2020 (n=91, 56%)	p-value
**Age, years**	61 [54-72]	63 [55-74]	0.54
**Female, n (%)**	22 (31)	22 (24)	0.36
**Body weight, kg**	80 [70-90]	83 [73-94]	0.17
**Height, cm**	174 [165-180]	174 [168-180]	0.99
**Body mass index, kg/m ^{2}**	26.0 [24.2-29.3]	27.7 [24.5-30.5]	0.24
**Diabetes mellitus, n (%)**	14 (19)	18 (20)	0.93
**Current smoker, n (%)**	29 (40)	42 (46)	0.46
**Hypercholesterolemia, n (%)**	57 (79)	75 (82)	0.62
**Hypertension, n (%)**	47 (65)	56 (62)	0.76
**Previous myocardial infarction, n (%)**	8 (11)	13 (14)	0.60
**Previous diagnosis of CCS, n (%)**	16 (22)	18 (20)	0.59
**Previous CABG, n (%)**	2 (3)	3 (3)	0.84
**Heart rate, bpm**	73 [65-90]	72 [60-92]	0.61
**Sinus rhythm, n (%)**	62 (86)	84 (92)	0.26
**Systolic blood pressure, mmHg**	134 [108-150]	128 [110-146]	0.50
**Diastolic blood pressure, mmHg**	73 [62-90]	78 [64-91]	0.44
**Killip class >I, n (%)**	20 (28)	30 (33)	0.42
**Peak troponin, x-fold increase of ULN**	268 [130-577]	279 [100-602]	0.97
**Total ischemic time, min**	165 [105-291]	258 [160-560]	**0.002**
**Door to balloon time, min**	40 [20-85]	43 [23-77]	0.81
**Culprit lesion, n (%)**			0.54
**RCA**	24 (33)	34 (37)	
**LAD**	35 (49)	43 (47)	
**LCX**	11 (15)	8 (9)	
**RI**	0 (0)	2 (2)	
**LM**	2 (3)	3 (3)	
**Bypass graft**	0 (0)	1 (1)	
**Pre-interventional TIMI flow 0, n (%)**	40 (56)	55 (60)	0.48
**Post-interventional TIMI flow 3, n (%)**	70 (97)	75 (82)	**0.004**

Abbreviations: CCS = Chronic coronary syndrome; CABG = Coronary artery bypass graft; ULN = Upper limit of normal; RCA = Right coronary artery; LAD = Left anterior descending artery; LCX = Left circumflex artery; RI = Ramus intermedius; LM = Left main coronary artery; TIMI = Thrombolysis in Myocardial Infarction.

eTable 2: Differences in clinical characteristics before and since March 16, 2020

	**Before March 16, 2020 (n=100, 61%)**	**From March 16, 2020 (n=63, 39%)**	**p-value**
**Age, years**	61 [54-74]	64 [56-74]	0.45
**Female, n (%)**	31 (31)	13 (21)	0.15
**Body weight, kg**	80 [70-90]	85 [73-97]	**0.04**
**Height, cm**	174 [165-180]	174 [168-180]	0.80
**Body mass index, kg/m ^{2}**	26.0 [24.2-29.1]	27.8 [24.6-30.8]	0.09
**Diabetes mellitus, n (%)**	18 (18)	14 (22)	0.48
**Current smoker, n (%)**	42 (42)	29 (46)	0.59
**Hypercholesterolemia, n (%)**	79 (79)	53 (84)	0.36
**Hypertension, n (%)**	65 (65)	38 (60)	0.73
**Previous myocardial infarction, n (%)**	15 (15)	6 (10)	0.52
**Previous diagnosis of CCS, n (%)**	26 (26)	8 (13)	0.12
**Previous CABG, n (%)**	3 (3)	2 (3)	0.94
**Heart rate, bpm**	72 [63-90]	74 [60-97]	0.98
**Sinus rhythm, n (%)**	87 (87)	59 (94)	0.35
**Systolic blood pressure, mmHg**	132 [110-150]	125 [107-152]	0.43
**Diastolic blood pressure, mmHg**	74 [63-90]	80 [64-94]	0.38
**Killip class >I, n (%)**	29 (29)	21 (33)	0.47
**Peak troponin, x-fold increase of ULN**	265 [108-577]	287 [108-602]	0.60
**Total ischemic time, min**	185 [115-393]	265 [160-505]	**0.03**
**Door to balloon time, min**	40 [21-72]	43 [23-94]	0.35
**Culprit lesion, n (%)**			0.14
**RCA**	37 (37)	21 (33)	
**LAD**	46 (46)	32 (51)	
**LCX**	12 (12)	7 (11)	
**RI**	0 (0)	2 (3)	
**LM**	5 (5)	0 (0)	
**Bypass graft**	0 (0)	1 (1)	
**Pre-interventional TIMI flow 0, n (%)**	54 (54)	41 (65)	0.18
**Post-interventional TIMI flow 3, n (%)**	95 (95)	50 (79)	**0.004**

Abbreviations: CCS = Chronic coronary syndrome; CABG = Coronary artery bypass graft; ULN = Upper limit of normal; RCA = Right coronary artery; LAD = Left anterior descending artery; LCX = Left circumflex artery; RI = Ramus intermedius; LM = Left main coronary artery; TIMI = Thrombolysis in Myocardial.
